# High Resolution Fluorescence Imaging of Cancers Using Lanthanide Ion-Doped Upconverting Nanocrystals

**DOI:** 10.3390/cancers4041067

**Published:** 2012-10-22

**Authors:** Rafik Naccache, Emma Martín Rodríguez, Nicoleta Bogdan, Francisco Sanz-Rodríguez, Maria del Carmen Iglesias de la Cruz, Ángeles Juarranz de la Fuente, Fiorenzo Vetrone, Daniel Jaque, José García Solé, John A. Capobianco

**Affiliations:** 1 Department of Chemistry and Biochemistry, Concordia University, Montreal H4B 1R6, Canada; E-Mails: r_naccac@live.concordia.ca (R.N.); emm_mart@live.concordia.ca (E.M.R.); nbogdan@alcor.concordia.ca (N.B.); 2 Departamento de Biología, Facultad de Ciencias, Universidad Autónoma de Madrid, Madrid 28049, Spain; E-Mail: francisco.sanz@uam.es (F.S.-R.); angeles.juarranz@uam.es (A.J.F.); 3 Departamento de Fisiología. Facultad de Medicina, Universidad Autónoma de Madrid, Madrid 28029, Spain; E-Mails: mc.cruz@uam.es; 4 Institut National de la Recherche Scientifique-Énergie, Matériaux et Télécommunications, Université du Québec, Varennes J3X 1S2, Canada; E-Mail: vetrone@emt.inrs.ca; 5 Departamento de Física de Materiales, Universidad Autónoma de Madrid, Madrid 28049, Spain; E-Mail: daniel.jaque@uam.es

**Keywords:** cancer, fluorescence imaging, lanthanide-doped nanocrystals, upconversion, targeting

## Abstract

During the last decade inorganic luminescent nanoparticles that emit visible light under near infrared (NIR) excitation (in the biological window) have played a relevant role for high resolution imaging of cancer. Indeed, semiconductor quantum dots (QDs) and metal nanoparticles, mostly gold nanorods (GNRs), are already commercially available for this purpose. In this work we review the role which is being played by a relatively new class of nanoparticles, based on lanthanide ion doped nanocrystals, to target and image cancer cells using upconversion fluorescence microscopy. These nanoparticles are insulating nanocrystals that are usually doped with small percentages of two different rare earth (lanthanide) ions: The excited donor ions (usually Yb^3+^ ion) that absorb the NIR excitation and the acceptor ions (usually Er^3+^, Ho^3+^ or Tm^3+^), that are responsible for the emitted visible (or also near infrared) radiation. The higher conversion efficiency of these nanoparticles in respect to those based on QDs and GNRs, as well as the almost independent excitation/emission properties from the particle size, make them particularly promising for fluorescence imaging. The different approaches of these novel nanoparticles devoted to “*in vitro*” and “*in vivo*” cancer imaging, selective targeting and treatment are examined in this review.

## 1. Introduction

Cancer is one of the leading causes of mortality in the World. Each year more than ten million people are diagnosed with this disease. While cancer cells are localized in a tissue, they can spread to distant sites within the body through a process known as metastasis. This has been one of the major challenges and has created numerous hurdles in finding a cure for cancer. Common cancer treatments include chemotherapy, radiation, therapy and surgery; however, these therapies are far from ideal and may not necessarily rid the patient of the cancer. In current cancer chemotherapies, nonspecific systemic distribution of antitumor agents through passive targeting, inadequate drug concentrations reaching the tumour site, as well as intolerable cytotoxicity, limit the therapeutic response and results in the eventual development of drug (or multiple drugs) resistance, which is a major problem when treating the cancer [1,2,3]. Radiation and surgery are more localized but may not be suitable in cases where the cancer has metastasized throughout the body.

Hence there exists a need to develop novel approaches for early cancer detection and effective therapy, and thus contributing significantly to improving patient survival and care. In this respect, different variants are continuously being developed including several ligand-targeted therapeutic strategies (such as immunotoxins, radio-immunotherapeutics and drug immunoconjugates) to bring forth improvements on conventional chemotherapeutic drugs and photodynamic therapy (PDT), which demonstrate variable results [2,4].

Nanotechnology has currently emerged as a new field of multidisciplinary research, implicating disciplines such as biology, chemistry, physics, engineering and medicine. Medical applications of nanotechnology have given place to a new science, presently known as nanomedicine. This new era in medicine has become very promising with a large potential in personalized oncology (cancer detection, diagnosis and therapy) [5].

Ever since scientists first pondered the idea of a nano-sized particle, nanoscience and nanotechnology have attracted a significant interest with the promise to revolutionize technologies, be it through innovation and invention, or through the modification and improvement of existing ones. In fact, new advances in nanoscience are being realized on a daily basis, and nanoparticles have already found integration in various industries such as textiles, automotive and glass to name a few, and even as potentially novel means for environmental clean-up to capture heavy metals, which are detrimental to ecosystems [5,6,7,8,9].

While nanoparticles have been and will undoubtedly be integrated in a multitude of future applications and technologies, of pertinent interest for this review, are the luminescent nanoparticles. This stems from their potential to be used as nanoprobes, capable of performing a multitude of tasks ranging from imaging to collection of diagnostic information and delivery of therapeutic agents (*vide infra*). Several types of luminescent nanoparticles have been the subject of meticulous investigation in the literature. In fact one such class of luminescent nanoparticles are quantum dots (QDs) [10,11,12,13,14,15,16,17,18]. Quantum dots represent the most extensively-studied luminescent nanoparticles, where a significant focus has recently been aimed towards biological applications and potential integration in imaging and diagnostics. The use of QDs does however raise numerous challenges associated with the safety and underlying toxicity especially for those QDs prepared using heavy metal elements such as CdSe, or CdTe [19]. In addition, imaging techniques using QDs usually rely on the use of ultraviolet (UV) light to excite the fluorescent label resulting in autofluorescence whereby both the analyte and the surrounding environment are excited showing multiple emissions and hence limited detection sensitivity. Moreover, research has shown that exposure to UV light can have adverse effects on cellular functions [20,21,22]. This effect is not observed using near-infrared (NIR) excitation light, which also offers a greater penetration depth in tissue systems relative to its UV counterpart [23]. This is due to the lower degree of scattering encountered when using NIR light, as scattering is dependent on the inverse of the excitation wavelength λ raised to the power of 4 (1/λ^4^) (Rayleigh scattering). This can allow for imaging, diagnostics and therapeutics far into the subcutaneous regions of the body. It is in this regard that there has been an ever increasing interest in another class of luminescent nanoparticles namely lanthanide (Ln^3+^)-doped nanoparticles, which have the ability to undergo a process known as upconversion. These nanoparticles allow for the conversion of low energy light such as NIR to high energy UV or visible light (blue, green, red for example) [24]. Unlike other materials such as gold nanorods or QDs, which can be excited using two-photon absorption (TPA), excitation in these upconverting Ln^3+^-doped nanoparticles (UCNP) does not occur *via* “virtual” excited states; it in fact occurs through excitation of the Ln^3+^ ions *via* “real” electronic states of defined (relatively long) lifetimes. Hence, high power, ultrafast, expensive laser setups are not required for efficient excitation but can be achieved using low-powered readily available solid-state laser diodes. The potential for excitation using NIR offers several advantages, as previously discussed, especially for imaging (*in vitro* and *in vivo*) and biomedical applications giving a distinct advantage over conventional fluorescent labels.

The interest in Ln^3+^-doped UCNPs even stems from beyond the potential to use them as luminescent biolabels. In fact, significant efforts are currently being undertaken to investigate the potential of using them as multi-modal systems capable of carrying out a multitude of tasks namely the harvesting of valuable diagnostic information, imaging, as well as delivery and therapeutics. In essence, these UCNPs are being envisaged as multimodal nanovectors, which through relatively simple surface modifications, can be functionalized to target specific analytes and receptor/cellular sites in biological systems. 

A significant focus in UCNP research has recently been directed towards cancer with an emphasis on developing nanoparticle conjugates, which can precisely image location of malignant cells by specifically seeking out tumor tissues. More importantly, current research has targeted an early diagnosis paradigm where cancerous tissue can be identified relatively early to allow for early intervention and improve the chances for successful treatment and patient survival rate. Nanoparticle surfaces can be readily manipulated in order to conjugate chemical entities. In that sense, specific and active targeting of overexpressed receptors (folates for example) on cancer cells can be achieved by functionalizing the appropriate molecule to the UCNP surface. Surface functionalization is not limited to one type of molecule, meaning molecules bearing various functional groups can be conjugated to the UCNP surface using well established bioconjugate chemistry techniques. It follows that UCNPs have become an interesting tool and offer a novel means to approach a treatment to a serious and life-threatening disease such as cancer.

This review aims to summarize the progress achieved to date regarding the use of luminescent UCNPs for *in vitro* and *in vivo* imaging of cancer cells.

## 2. Fluorescence Properties

**Figure 1:**
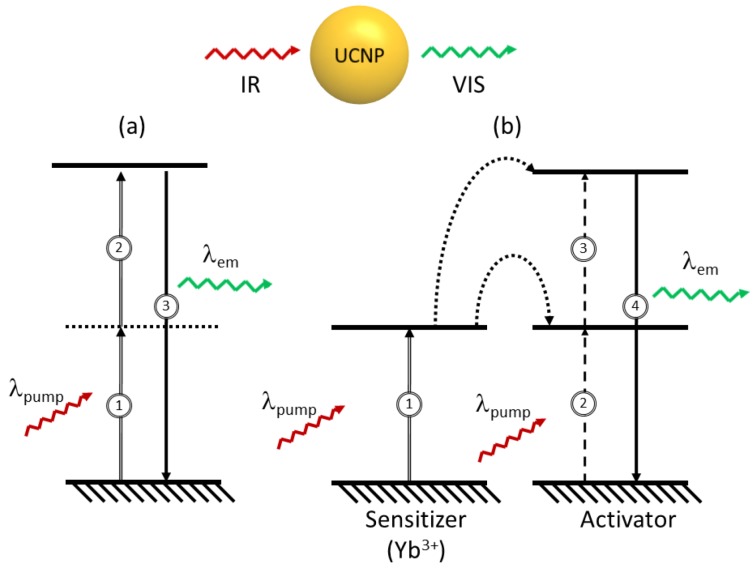
Principal mechanisms for upconversion (**a**) excited state absorption, (**b**) energy-transfer.

In a typical case, when a medium fluoresces as a consequence of excitation with a source of light, the wavelength of the fluorescence emission is usually longer than that of the excitation source. However, under certain circumstances, light may be emitted with wavelengths shorter than those of the light generating the excitation. These processes are possible *via* excitation mechanisms, which involve more than one absorbed photon per single emitted photon and are known as upconversion. The simplest example of an upconversion process is illustrated in Figure 1a, and takes place *via* sequential absorption of pump photons. In this multistep mechanism, a first absorption process excites an ion in the ground state to some metastable excited level (that can be a real or a virtual level). In the second step, further absorption may take place raising the ion to a higher excited state . Finally, the ion relaxes back to the ground state with the emission of a photon of light equivalent to the energy gap between the excited and the ground states.

Lanthanide ions are among the most efficient systems capable of producing upconversion luminescence; hence emission in the visible can be achieved following excitation in the near-infrared (NIR) regime [24,25]. Although upconversion emission can be theoretically expected from most lanthanide ions, visible optical emissions under low pump power intensities (ca. 10 W/cm^2^) are only generated by ions, which possess equally spaced energy levels facilitating photon absorption and energy transfer steps involved in upconversion processes. Tripositive erbium (Er^3+^), thulium (Tm^3+^), and holmium (Ho^3+^) are examples of ions possessing these “ladder-like” levels and are typically selected as “activator” (or acceptor) ions for the majority of UCNP systems [24,26,27,28,29,30,31,32,33].

To enhance upconversion efficiency, tripositive ytterbium (Yb^3+^) is frequently used as a “sensitizer” (or donor) in combination with the activator ion. The Yb^3+^ absorption band, located at *circa* 980 nm, has a larger absorption cross-section relative to that of the other lanthanide ions (Er^3+^, Tm^3+^ or Ho^3+^) and its emission band matches well with many emissions of typical upconverting lanthanide ions (Ln^3+^) thereby facilitating efficient energy transfer from the Yb^3+^ ion to other Ln^3+^ ions. A basic scheme of the absorption and emission processes in a typical upconverting nanoparticle comprised of sensitizer and activator ions is presented in Figure 1b. The sensitizer ion (typically Yb^3+^) is raised to an intermediate excited state *via* an incoming pump photon ①. A non-radiative energy transfer to a neighbouring activator ion will raise the activator to a resonant intermediate excited state ②. A second non-radiative energy transfer may occur further raising the activator ion to a higher excited state ③. This is followed by visible emission of the activator ion while the sensitizer Yb^3+^ ion returns to the ground state ④. Hence, the sensitizer and the activator interact to excite the activator to the higher energy level from which the visible emission takes place. The efficiency of this process is highly dependent on the structure and symmetry of the host crystal. Materials with low-energy phonon vibrations have been shown to be among the strongest emitters. The importance of the symmetry has been demonstrated in the fact that the hexagonal phase of NaYF_4_:Er^3+^, Yb^3+^ (or NaGdF_4_:Er^3+^, Yb^3+^) crystals possess a higher efficiency relative to their cubic counterparts [34]. Numerous and different emission colors may be obtained by the variation of the type and concentration of the sensitizers and activators [26,35].

**Figure 2:**
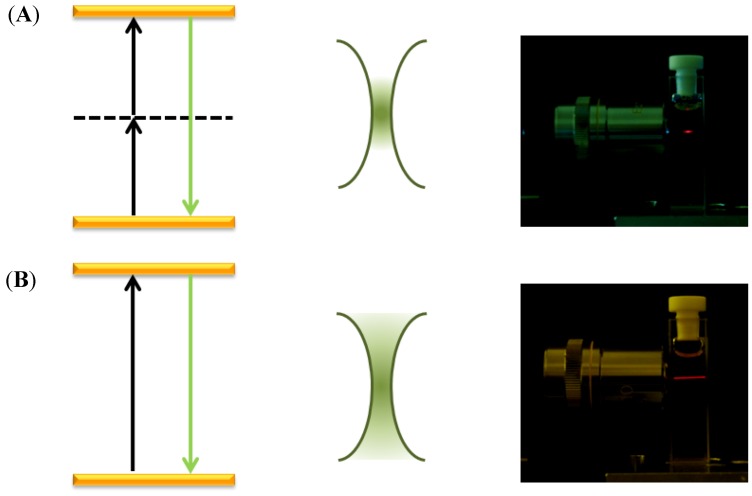
Schematic explanation of the increment of the spatial resolution achieved by using (**A**) two-photon upconversion processes when compared to (**B**) a single photon absorption process. The energy level represented by the solid line may represent either a real or a virtual intermediate state (such as in the case for quantum dots).

The non-linear nature of the excitation process of upconversion makes possible for the use of UCNPs as luminescent markers for upconversion imaging [36]. Traditional two-photon excited imaging relies on the use of high-power NIR lasers focused to a spot size in which an efficient two-photon excitation of the fluorophore can occur. As the excitation probability of the fluorophores shows a quadratic dependence on the incident pump power, the excited sample volume is restricted to the focal volume of the beam, so that the spatial resolution may be improved (Figure 2) [37]. Collection efficiency is also higher than that in other high-resolution microscopy techniques as two-photon excitation fluorescence microscopy is based on limiting the excited volume instead of limiting the observation area. As the excitation is achieved using NIR luminescence, two-photon excited imaging is particularly suitable for studying deep tissue systems. In addition to the higher penetration depth in tissues, the NIR radiation is less phototoxic relative to UV radiation traditionally used in bioimaging experiments. On the other hand, the localization of the two-photon excitation volume to the focal plane further minimizes the volume in which tissue damage may occur to a sub-femtolitre volume. Both aspects result in a dramatic increase in viability of biological specimens.

Organic fluorophores that have been traditionally used for luminescent bioimaging present several drawbacks; for instance, they have broad emission spectra unsuitable for multiplex biolabeling, require UV light for proper excitation, and often suffer from photodegradation on exposure to external illumination. These drawbacks can be mediated by using inorganic nanoparticles such as QDs, gold nanorods (GNRs) and UCNPs. In particular, high-resolution optical images have been obtained by using quantum dots nanoparticles (QDs), and gold nanorods (GNRs). Both types of nanoparticles (QDs and GNRs) share the capability of converting NIR light to visible with the UCNPs; however, they do suffer from numerous drawbacks. In the case of QDs, UV is typically the principal source of excitation, which is less advantageous in comparison to NIR. Furthermore, the use of QDs for biological detection is limited by several factors, mainly their potential toxicity, as they include in their components heavy metals (e.g., Cd) [38,39]. Several non-toxic based QDs based on ZnS have been proven for biological imagining, however, they still present the limitation of UV excitation [40,41,42,43]. Intermittent emission (blinking) also limits their use for labeling individual biological molecules [38]. The use of GNRs may be attractive in therapeutic applications [44]; however, it can cause damage to the biological specimens due to localized heating of the GNRs, which result from the plasmon resonance with the pump beam.

Upconverting nanoparticles present an interesting alternative to QDs and GNRs. To date, no significant toxic effects have been reported in cellular studies using UCNPs [45,46,47,48,49]. In fact little to no toxicity has been reported in *in vivo* studies using the mouse model. As the nanoparticles typically contain a large number of Ln^3+^ dopant ions, they do not blink under continuous irradiation of a NIR laser [50,51], which is contrary to what has been observed for QDs. Upconversion occurs within the host crystal; it is therefore less affected by the chemical and biological environments in comparison to the emission of QDs and GNRs. Furthermore, no heating effects have been observed due to the use of UCNPs in imaging experiments of cancer cells [52].

**Figure 3:**
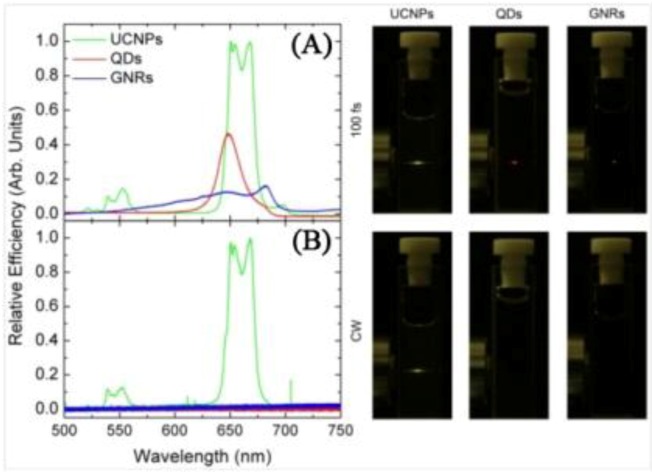
Left Side. (**A**) Multiphoton relative luminescence signal generated by the QD, GNR, and UCNP solution as obtained under fs laser excitation at 800, 830 and 980 nm, respectively. Pump intensity was 1 MW/cm^2^; (**B**) Multiphoton relative luminescence signal generated by the QD, GNR, and UCNP solution as obtained under cw laser excitation at 800, 830 and 980 nm, respectively. Pump intensity was 1 MW/cm^2^. Right Side: Photos corresponding to the naked eye observations of the fluorescent samples. The pump intensity was kept the same in both cases (CW and 100 fs excitation) ([53], reproduced by permission of The Optical Society of America).

The principal advantage of UCNPs is that they present higher NIR-to-visible conversion efficiency relative to both QDs and GNRs as can be seen in Figure 3 [53] as their emission is based on physically existing states. The probability of two-photon absorption is proportional to the square of the incident laser intensity, so in general the use of very intense laser pulses is necessary (in general ultra-short pico- or femto-second pulsed lasers, so the average power is low and thermal damage to the sample can be minimized). The superior efficiency of UCNPs makes possible of the use of low intensities (1–10^3^ W/cm^2^) and so inexpensive continuous wave lasers for imaging processes, instead of the high-intensities (10^6^–10^9^ W/cm^2^) associated to ultra-fast pulsed laser sources for the generation of a simultaneous two-photon excitation process. Hence, this reduces the elevated powers needed at the focal volume where photochemical interactions occur, and minimizes the damage brought about to biological samples. Upconverting nanoparticles have the important advantage of being photoexcitable in the near infrared (NIR) (most often at around 980 nm) in the region known as the biological optical window. In this region the auto-absorption of any biological matter is quite weak (with the exception of water where absorption is moderately weak) thereby reducing, to virtually zero, any background absorption and autofluorescence. In addition, commercially available diode laser systems are well matched to the absorption of Yb^3+^ at 980 nm, providing an ideal and inexpensive excitation source for UCNPs.

From the image analysis perspective, UCNPs present multiple advantages relative to other fluorophores. Firstly, the emission bands of the lanthanide ions are quite narrow thus enabling their easy separation, leading to increased selectivity of the imaging assay. Secondly, different Ln^3+^ ions or combination of Ln^3+^ ions will emit at different visible wavelengths upon excitation with 980 nm, suggesting that multiple analytes can be detected simultaneously by simply changing the dopant ion(s) in the UCNP. Thirdly, the long fluorescent lifetimes of most Ln^3+^-doped materials (μs to ms range) renders them highly suitable for time-gated detection applications since any inherent fluorescence from biological systems, for example proteins, with natural fluorescence is in the 1–10 ns range, can be eliminated. Finally, the wavelengths of the Ln^3+^-doped UCNP fluorescence is not particle-size dependent and each Ln^3+^ dopant ion possesses unique emission lines thus allowing for multiplexing capabilities.

## 3. Synthesis, Surface Modification and Bioconjugation

In recent years, several research groups have prepared UCNPs, using various synthetic techniques, capable of producing intense luminescence in the UV to visible regions following NIR excitation [49,54,55,56]. Among the various types of UCNPs, NaYF_4_ (and the isostructural NaGdF_4_) is known to be one of the most efficient UCNP hosts due to its intrinsically low lattice phonon energies [33,57]. The crystal structure of NaYF_4_ manifests itself in two forms, namely the cubic (α) and hexagonal (β) phases. The hexagonal β-phase of NaYF_4_ has three cation sites, the first accommodating yttrium ions and the second accommodating both yttrium and sodium ions with C_3_ symmetry. The third site accommodates sodium ions and possesses C_s_ symmetry. Previous investigations have shown that the luminescence intensity signal of hexagonal β-NaYF_4_ is at least one order of magnitude greater than that of the cubic α-NaYF_4_ [34]. NaYF_4_/NaGdF_4_ co-doped with trivalent rare earth ions (Ln^3+^) have been the subject of many investigations for use in biological applications. Such applications include fluorescence resonance energy transfer (FRET) assays, DNA detection, diagnostics, targeting, drug delivery, *in vivo*/*in vitro* cancer targeting and imaging as well as photodynamic therapy [49,51,52,53,58,59,60,61,62,63,64,65,66,67,68,69,70,71,72,73,74,75,76]. Additionally, as a potential multimodal imaging probe, NaGdF_4_ has been evaluated for use in both simultaneous fluorescence and magnetic resonance imaging (MRI) [51]. We present an overall review of the most prominent synthetic approaches to prepare highly luminescent and water-dispersible UCNPs (through direct synthesis or surface modification). Furthermore, strategies for surface chemical modifications useful for the introduction and conjugation of biomolecules at the surface of the UCNPs necessary for targeting and delivery are also discussed.

The hydro/solvothermal method is one common approach to prepare fluoride-based UCNPs. For this synthesis, NaCl, NH_4_F and lanthanide chlorides are used as the precursors to the UCNP formation [77]. This method uses a pressurized autoclave and relatively low reaction temperature conditions in comparison to the thermal decomposition technique (*vide infra*) [78]. The shape, the crystalline phase and the size of the nanoparticles are controlled using hydrophobic organic ligands such as oleic acid [79]. The hydrothermal synthesis is an effective and convenient method for the preparation of the pure hexagonal (β) phase of NaYF_4_ with controllable morphologies and architectures including plates, spheres and ellipses by varying the oleic acid surfactant concentration [77]. In the same approach, hexagonal-phase β-NaYF_4_:Yb^3+^, Er^3+^ UCNPs stabilised with cetyltrimethylammonuim bromide (CTAB) with controllable size and morphology were produced by Li *et al*. [33]. Many studies have reported that hydrophilic molecules such as polystyrene [80], ethylenediamine tetraacetic acid (EDTA) [81] or an amphiphilic surfactant such as poly(vinylpyrrolidone) (PVP) [82] may be used as chelating agents to enhance the stability, aqueous dispersibility and potential for functionalization of the fluoride-based UCNPs.

Polyol synthetic routes may also be used to prepare UCNPs. In fact, aqueously dispersible cubic (α) NaYF_4_:Yb^3+^, Er^3+^ and NaYF_4_:Yb^3+^, Tm^3+^ UCNPs, with a mean particle size of 17–30 nm showing multi-color upconversion emission for multiple labeling in biological detection, were obtained using polyol capping ligands [83]. The cubic-to-hexagonal phase transition may be carried out during the solvothermal process and results in an enhanced fluorescence intensities of the UCNP emissions. From one-step hydrothermal methods, water-dispersible polyethylenimine (PEI)-capped NaYF_4_:Yb^3+^, Er^3+^ or Tm^3+^ UCNPs were synthesized [60]. PEI offers several advantages as a capping ligand principally due to its low toxicity profile and potential for further functionalization due to the terminal NH_2_ groups. Zhang *et al*. demonstrated that PEI-capped UCNPs showed little toxicity especially to HT-29 colon cells following MTT assays, which showed a high cell survival rate. The surface of the PEI-capped NaYF_4_:Yb^3+^, Er^3+^ UCNPs synthesized using the hydrothermal method were subsequently conjugated with folic acid following a reaction between the carboxyl groups of folic acid and the amino groups of the PEI-capped UCNPs [60].

**Figure 4:**
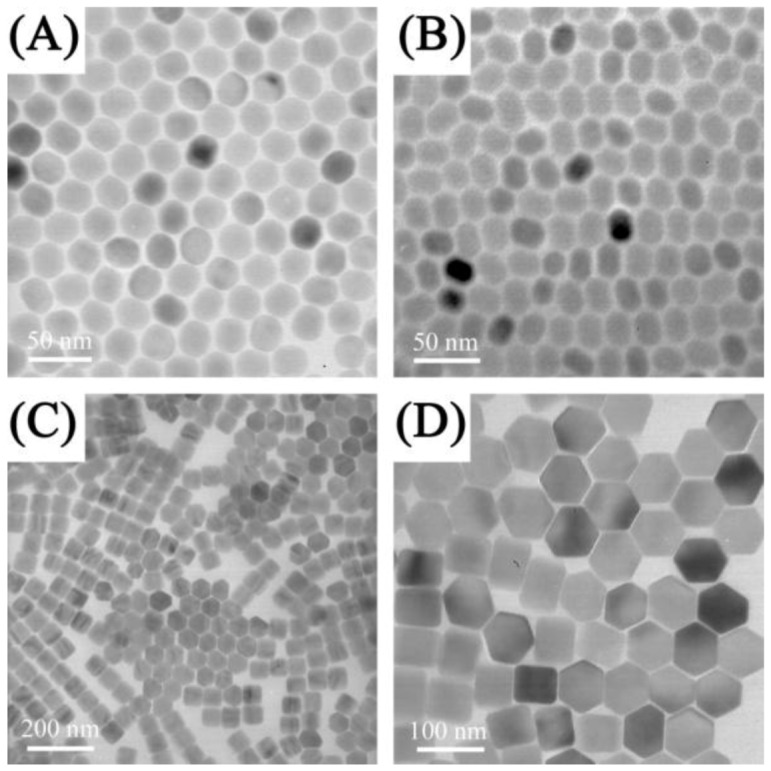
TEM images of NaYF_4_:Yb^3+^, Er^3+^nanospheres (**A**), nanoellipses (**B**), nanoplates (**C**, **D**)obtained by solvothermal methods ([85], reproduced by permission of the Institute of Physics).

A novel two-phase solvothermal approach was used for the synthesis of hydrophilic β-NaYF_4_:Yb^3+^/Er^3+^/Tm^3+^/Ho^3+^ with an average particle size of approximately 25 nm using lanthanide stearates as precursors [26]. The same group also reported the synthesis of water dispersible spherically-shaped NaYF_4_:Yb^3+^, Er^3+^ UCNPs stabilized with polyacrylic acid (PAA), polyvinylpyrrolidone (PVP), or polyethylene glycol (PEG) with a particle size distribution ranging from 25 to 45 nm using a one-step hydrothermal synthesis [84]. Recently, a user-friendly method previously developed by Li [85] (Figure 4) was used to synthesize NaYF_4_:Yb^3+^, Er^3+^/NaGdF_4_ core/shell UCNPs [86] coated with silica for *in vivo* optical and magnetic resonance imaging applications. In a modified hydrothermal microemulsion strategy, 6-aminohexanoic acid was used for the preparation of amine-functionalized UCNPs [65]. These UCNPs were conjugated *via* an amide linkage through folic acid-NHS conjugates.

**Figure 5:**
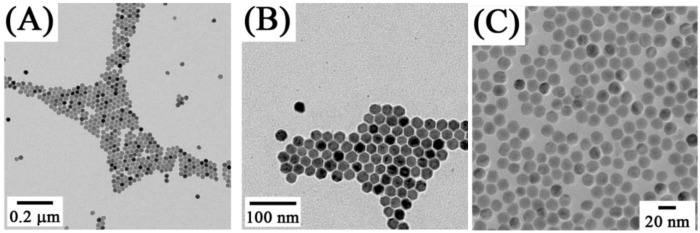
(**A**, **B**) Low-resolution TEM images of NaYF_4_: Yb^3+^, Er^3+^ sample showing uniformity of the particles [27] and (**C**) TEM image of oleic acid-capped NaGdF_4_:Yb^3+^ /Ho^3+^ obtained from thermal decomposition ([91], reproduced by permission of The American Chemical Society).

The thermal decomposition synthesis presents several advantages over other preparation methods, namely the strict control over the synthetic parameters and the formation of highly monodisperse and luminescent UCNPs [27,78,87,88,89,90]. In this method, the lanthanide oxides are first transformed to the trifluoroacetate form and the resulting precursors of the lanthanide organic acid salts are then decomposed, in the presence of a capping ligand (such as oleic acid) to produce hydrophobic UCNPs. Controlling the ratio of the precursors to the capping ligands, as well as the reaction temperature and time allows for a tailored synthesis of UCNPs with a variety of morphologies, sizes and phases. Using this method Capobianco’s group synthesized cubic phase NaYF_4_ UCNPs co-doped with the Er^3+^/Yb^3+^ or Tm^3+^/Yb^3+^ and hexagonal phase NaGdF_4_:Ho^3+^/Yb^3+^ with a particle size ranging from 15 to 27 nm [27,28,87,91] (Figure 5). In addition, diamond-shaped LiYF_4_:Tm^3+^/Yb^3+^ UCNPs were obtained using the same synthetic route. Capobianco’s group also reported on the synthesis of core/shell UCNPs and found that following shell formation, significant upconversion luminescence enhancement was observed. This was observed for the core/shell UCNPs comprised of NaGdF_4_:Ce^3+^, Tb^3+^ (core)/NaYF_4_ [92] and NaGdF_4_:Er^3+^, Yb^3+^ (core)/NaGdF_4_:Yb^3+^ [93]. Other groups have also reported on the synthesis of core/shell systems such as NaYF_4_:Yb^3+^, Er^3+^ (core)/NaGdF_4_ [86], which may provide a great potential as magnetic and upconverting fluorescent bimodal cancer probes. In this context, Ryu *et al*. recently reported on the synthesis of hexagonal phase NaGdF_4_:Yb^3+^, Er^3+^ (or Tm^3+^) UCNPs ranging in size from 10–40 nm in size, which show efficient upconversion and magnetic properties [94].

**Figure 6:**
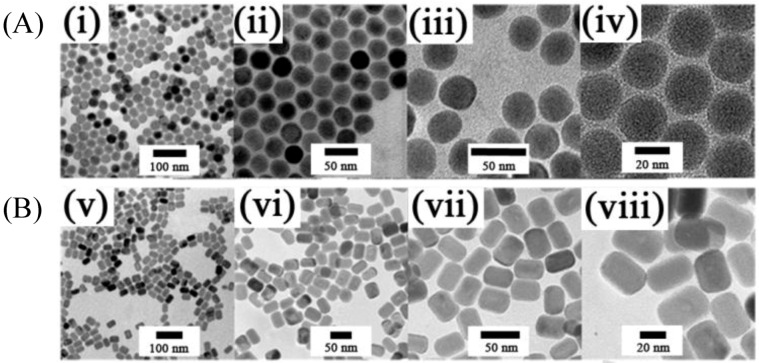
(i–viii) TEM images taken with different magnifications of: (**A**) β-NaYF_4_:Yb^3+^,Er^3+^ (Tm^3+^) [95] and (**B**) β-NaGdF_4_:Ce^3+^ (Yb^3+^) Ln nanorods (Ln:Tb, Eu, Dy, Sm, Er or Tm) UCNPs [96] synthesized by microemulsion in a thermo decomposition methods fromrare earth oleate as precursors(Reproduced by permission of The Royal Society of Chemistry).

Another thermal decomposition approach relies on the use of a microemulsion method. In this synthesis, the trifluoroacetate precursors (typically used in thermal decomposition) are replaced with NaF and rare earth oleates as precursors [95]. This approach is simple and requires no injection processes (used for the addition of the precursors to the reaction vessel). During the reaction no additional capping-reagents or surfactants are required to maintain size control as the sizes may be simply tuned by adjusting the concentration of NaF. Using this approach, oleate-capped β-NaYF_4_:Yb^3+^, Er^3+^ (or Tm^3+^) UCNPs showing high monodispersibility and particle sizes ranging from 18–45 nm have been synthesized (Figure 6). In the same procedure the up-conversion luminescence was enhanced by using a core/shell structure comprised of NaYF_4_:Yb^3+^, Er^3+^/NaYF_4_ (undoped). Moreover monodisperse, well-crystallized and multi-colored-emission oleate-capped NaGdF_4_:Ce^3+^(Yb^3+^), Ln^3+^ (Ln^3+^ = Tb^3+^, Eu^3+^, Dy^3+^, Sm^3+^, Er^3+^ or Tm^3+^) comprised of uniform short nanorods were also obtained [96].

The thermal decomposition method is an excellent synthetic procedure to prepare UCNPs; however, the main disadvantage lies in the fact that it requires high reaction temperatures (310–330 °C) and high-boiling organic solvents such as octadecene. Furthermore, while the UCNPs prepared *via* thermal decomposition have well defined morphologies and sizes, they are hydrophobic and require further modification for aqueous dispersibility. These hydrophobic UCNPs may be transformed into water-dispersible UCNPs *via* a ligand-exchange pathway with various hydrophilic polymers or through silica coating.

### 3.1. Strategies for Water Dispersibility

Different synthetic approaches with special emphasis on applications in biolabeling, targeting and imaging are discussed below and concern themselves with processes which allow for the transfer of hydrophobic nanoparticles to a hydrophilic medium.

To render hydrophobic oleate-capped nanoparticles water dispersible, one approach involves oxidation of the carbon-carbon double bond of the oleate ligand. The oxidation of the carbon-carbon double bonds of the oleate ligand, using the Lemieux-von Rudloff reagent [97], results in the formation of azelaic acid (C9), which renders the hydrophobic oleic acid-capped UCNPs, obtained by thermal decomposition methods, water dispersible. The carboxyl groups (-COOH) obtained at the surface of UCNPs may be further conjugated to biomolecules. Recently, a novel approach [98] was developed to obtain water-dispersible ligand-free upconverting lanthanide-doped NaYF_4_:Er^3+^,Yb^3+^ nanoparticles with high upconversion luminescence intensities in aqueous solutions. This approach was based on removing the oleate ligand from the surface of oleate-capped upconverting nanoparticles synthesized *via* thermal decomposition using a simple acid treatment process. It was shown that after removing the oleate, the surface charge of the Ln-UCNPs can be tuned through pH control where intense red emission was obtained at neutral pH using this strategy. These ligand-free upconverting nanoparticles were further functionalized with heparin and basic fibroblast growth factor (bFGF) molecules [99]. The heparin conferred stability and protected bFGFs from inactivation. The presence of bFGF at the surface of the nanoparticles assured affinity with the fibroblast growth factor receptors at the cell membrane. These heparin-bFGF functionalized nanoparticles showed biocompatibility with mammalian cells and were successfully used in upconversion fluorescence to imagine the HeLa epithelial cancer cells.

**Figure 7:**
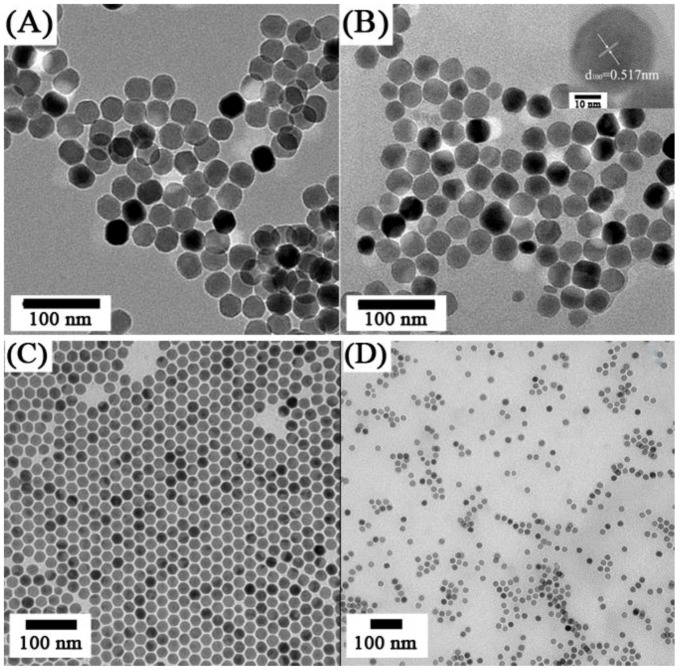
TEM images of oleic acid-UCNPs dispersed in (**A**) cyclohexane and (**B**) citrate coated-UCNPs dispersed in water—Inset: HRTEM image of single nanoparticle ([104],reproduced by permission of Elsevier) and (ii) TEM images of NaYF_4_:Yb^3+^, Er^3+^ (**C**) oleate-stabilized, (**D**) ligand-exchanged with PVP ([108], reproduced by permission of the Royal Society of Chemistry).

Alternative approaches to achieve water dispersibility for hydrophobic UCNPs include ligand exchange reactions using a number of different ligands such as poly(ethylene glycol) (PEG) [100,101], PEG-diacid [102], PEG-phosphate [103], sodium citrate [104], hexanedioic acid (HDA) [105], polyacrylic acid (PAA) [91,106,107] or polyvinylpyrrolidone (PVP) [106] (Figure 7).

**Figure 8:**
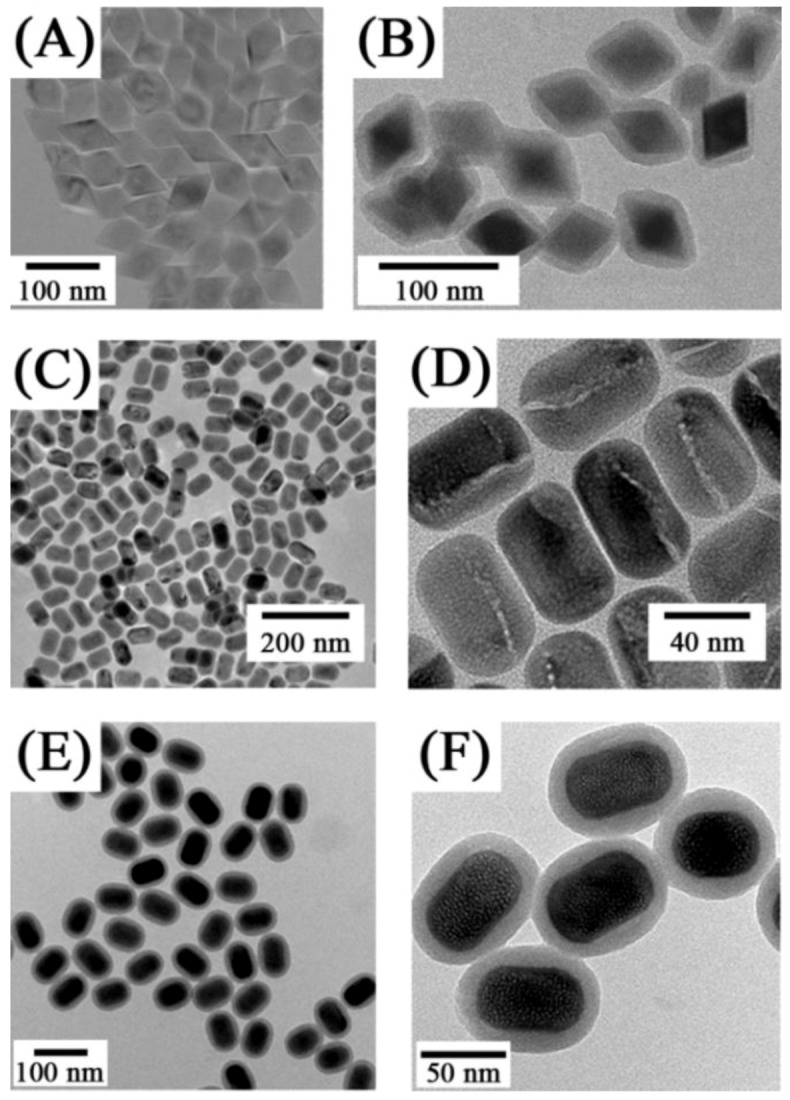
TEM images of LiYF_4_:Yb^3+^, Tm^3+^ nanocrystals ([28]) (**A**) before and (**B**) after silica coating as well as TEM images of NaYF_4_:Yb/Er up-conversion nanocrystals: (**C**, **D**) before and (**E**, **F**) after silica coating ([75], reproduced by permission of Wiley).

Other approaches rely on the use of hydrophobic van der Waals interactions, electrostatic layer-by-layer assembly or surface polymerisation for encapsulation of hydrophobic UCNPs with amphiphilic polymers or silanes (Figure 8) to obtain water-dispersible UCNPs [54]. These encapsulated-UCNPs possess functional groups, which allow for further biological modifications. For example, the hydrophobic hexagonal phase of NaYF_4_:Yb^3+^, Er^3+^ (Tm^3+^) core and NaYF_4_:Yb^3+^, Er^3+^ (Tm^3+^)/NaYF_4_ NP core/shell systems can be rendered hydrophilic using a layer of amphiphilic poly(acrylic acid) (PAA) polymer as reported in the work of Yi *et al*. [109]. In another example, the hydrophobic UCNPs synthesized *via* thermal decomposition were rendered water dispersible following labeling with octylamine modified poly(acrylic acid) polymer conjugated, using EDAC, with an amino-functionalized poly(ethylene glycol) PEG (OA-PAA-PEG) molecule [110]. The polyethylene glycol (PEG) groups present on the surface of UCNPs confer aqueous dispersibility and biocompatibility. In a recent work, the PEG amphiphilic polymer was also used to render the hydrophobic NaYF_4_:Yb^3+^, Er^3+^ UCNPs water dispersible [63]. These PEGylated UCNPs were labeled with folic acid (FA) by conjugating the amine-functionalized PEG-NH_2_ with activated folic acid. The FA-PEG-UCNPs were further loaded with a chemotherapy drug molecule, doxorubicin (DOX), by simple physical adsorption *via* a supramolecular chemistry approach.

### 3.2. Surface Modification and Functionalization for Biological Applications

**Figure 9:**
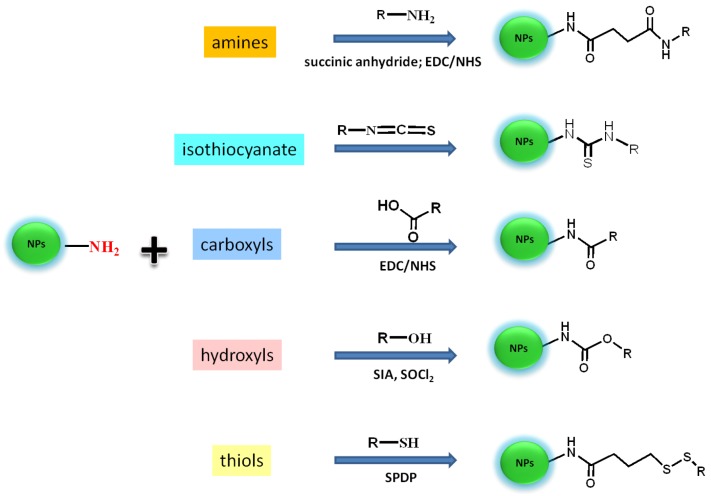
Bioconjugation schemes for the attachment of bio(molecules) (R) presenting different target sites (amines, carboxyl, hydroxyl, thiols, isothiocyanate) onto the surface of amine-coated-UPNPs. Reagents: 1-ethyl-3-(3-dimethylaminopropyl) carbodiimide hydrochloride (EDC), succinimidyl ester (NHS), succinimidyl iodo acetate (SIA), *N*-succinimidyl 3-(2-pyridyldithio) propionate (SPDP).

**Figure 10:**
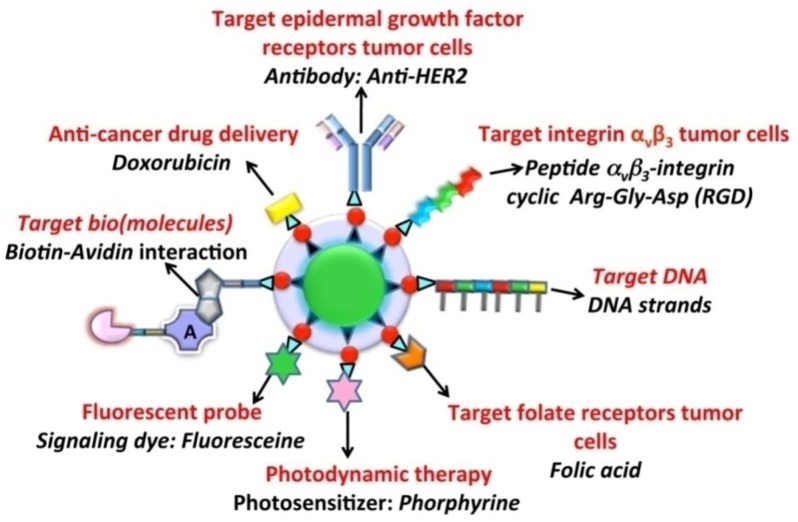
Different functionalization approaches used for UCNPs in order to provide a targeting or therapeutic function.

As shown above, a multitude of approaches have been employed for the preparation of UCNPs amenable to functionalization for biological applications. For such applications, it is highly desirable to prepare UCNPs, which are dispersible in bio-relevant media, with a surface that can be appropriately functionalized with targeting molecules such as, antibodies, peptides, cancer specific receptor-recognition molecules, drugs, *etc*. [111]. Numerous investigations have shown that modification of the UCNP surface with hydrophilic molecules allows for the introduction of reactive groups such as amino (NH_2_) or carboxylic (COOH) groups and may improve stability as well as the dispersibility of the particles in biological media. These groups allow for covalent binding with specific diagnostic and therapeutic (bio)molecules, fluorophores as well as targeting moieties as previously mentioned. Covalent binding is preferred in biological applications so as to avoid ligand desorption from the nanoparticle surface. The most important chemical modification approaches using amine-terminated UCNPs for covalent binding with biomolecules are presented in Figure 9. Biomolecules are usually pre-activated to introduce the appropriate chemical functionality, which allows for bond formation to occur with the UCNP-NH_2_ surface. The most common activation chemistry relies on the preparation of a *N*-hydroxysuccinimide (NHS) ester that can be formed by the reaction of a carboxylate with NHS in the presence of 1-ethyl-3-(3-dimethylaminopropyl)carbodiimide (EDAC). The latter mediates the formation of an amide linkage between a carboxylate group and an amine [112]. Other strategies for biolabelling are based on the strong non-covalent biological interaction between avidin/streptavidin (or variations thereof) and biotin Figure 10. For example, streptavidin may be covalently coupled with antibodies or enzymes through EDAC; the biotin-streptavidin combination has been investigated in many biotinylated nanoparticle systems [112].

## 4. *In Vitro* and *in Vivo* Imaging

**Figure 11:**
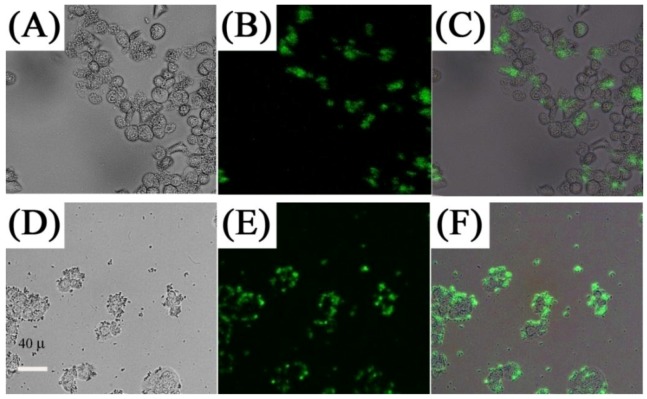
*In vitro* imaging using folic acid modified PEI/NaYF_4_ nanoparticles The top row shows the bright field (**A**), confocal (**B**), and superimposed images of live human ovarian carcinoma cells (OVCAR3) (**C**) and the bottom (**E**, **F**) human colonic adenocarcinoma cells (HT29) ([71], reproduced by permission of Elsevier).

One of the first demonstrations of UCNPs as two-photon excited fluorescent imaging probes was published by Chatterjee *et al*. [71] in 2008. In this seminal work, the authors demonstrated that NaYF_4_:Er^3+^, Yb^3+^ UCNPs coated with polyethylenimine (PEI) and conjugated with folic acid could be used to target human HT29 adenocarcinoma cells and human OVCAR3 ovarian carcinoma cells. These two cell lines were used since they are known to express abnormally high levels of folate receptors on their surfaces. The folic acid conjugated UCNPs were incubated for different times and after washing, the visible upconversion fluorescence of the UCNPs attached to the cell surface was easily observed following excitation with 980 nm NIR radiation (Figure 11). Moreover, it should be noted that luminescence from the UCNPs could be observed within an hour; however, cellular uptake of the nanoparticles was much slower and required approximately 24–48 h of incubation. Also of note from this work, the authors injected Wistar rats with the PEI-coated UCNPs at a depth of approximately 10 mm and visible fluorescence was clearly observed.

Following this first report on cellular imaging using UCNPs, the potential of lanthanide-doped UCNPs became apparent. From 2008–2011, a stark increase in publications concerning imaging using UCNPs was observed in the literature. A significant number of publications placed a strong emphasis on the imaging capabilities at the cellular level. These works also focused on the imaging of cancer cells. A wide variety of cell lines was investigated encompassing breast, colon, ovarian, skin, pancreatic, ocular, and brain cancer cell lines *(vide infra)*. The vast majority of these works focused on the synthesis of Ln^3+^-doped UCNPs, many of which required post synthetic modification to achieve water dispersibility, and their surface functionalization to target the cancer cells. Moreover, most of the works reported focused on fluoride-based UCNPs, including NaYF_4_, NaGdF_4_ and LaF_3_ with some works reporting the use of an oxide host, namely the well-studied Y_2_O_3_ [113,114] During the imaging experiments, the binding/uptake of the UCNPs into the various cell lines occurred through non-specific means [26,31,46,47,52,64,73,75,76,103,104,115,116,117].

**Figure 12:**
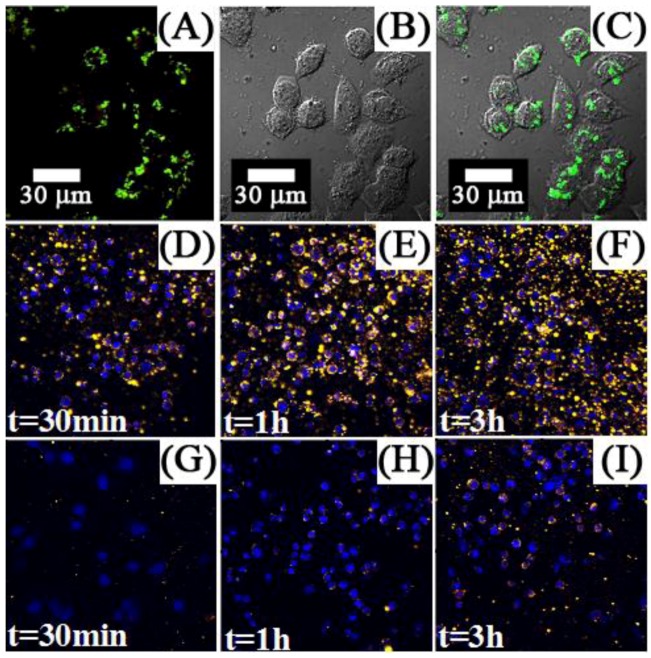
First row: luminescence (**A**), bright-field (**B**) and overlap of both images (**C**), of KB cells cultured with UCNPs-Ad/β-CD ([123], reproduced by permission of the Royal Society of Chemistry). Second and third rows: Fluorescence imaging of MB49 cells cultured with: carbonized glucose-coated UCNPs (**D**), (**E**) and (**F**); silica-coated UCNPs (**G**), (**H**) and (**I**) ([120], reproduced by permission of the Institute of Physics).

**Figure 13:**
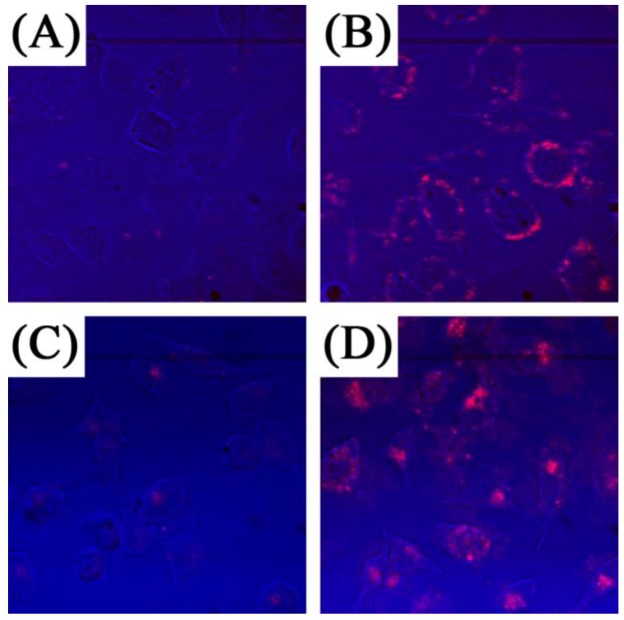
Confocal images of Panc 1 cells treated with up-conversion NPs (**A**,**B**) and down-conversion NPs (**C**, **D**). (**A**) and (**C**) show non-bioconjugated nanophosphors, (**B**) and (**D**) show cells targeted with nanophosphors conjugated with anti-claudin 4 and anti-mesothelin, respectively ([124], reproduced by permission of Wiley).

**Figure 14:**
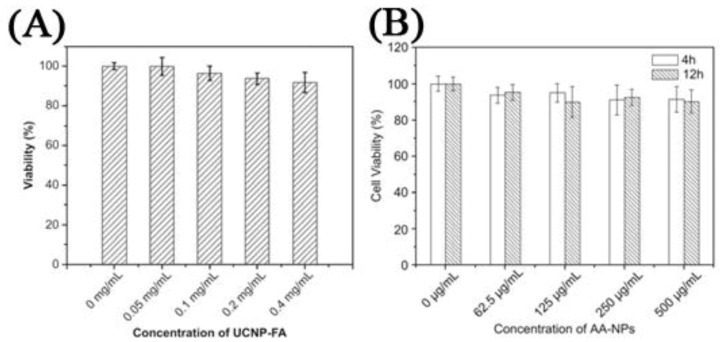
Left: Cell viability estimated by MTT proliferation tests for different incubation concentrations of: (**A**) HeLa cells incubated with Folic Acid-UCNPs [65]; (**B**) KB cells cultured in the presence of citrate capped-NPs ([125], reproduced by permission of Elsevier).

**Figure 15:**
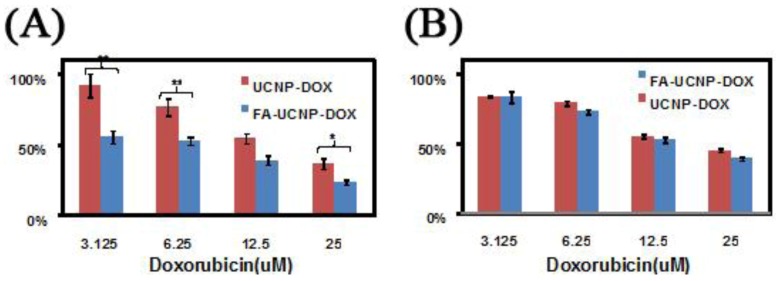
Cell viability data of FR positive KB cells (**A**) and negative HeLa cells (**B**) treated with FA-UCNP-DOX and UCNP-DOX ([63], reproduced by permission of Elsevier).

In contrast, several works investigated the use of targeting molecules as a means for specific binding, which can lead to efficient cellular uptake and enhanced imaging within the cell walls and organelles. Several targeting molecules have been reported including organics, such as folic acid, β-cyclodextrins and carbonized glucose (Figure 12) [63,65,71,118,119], or peptides and antibodies such as RGDyK, cyclic RGD and anti-Claudin 4 to name a few (Figure 13) [26,66,70,72,120]. One of the most important findings in these works was related to the specific uptake of the UCNPs in the cell in comparison to non-surface functionalized UCNPs. The difference in emission intensity following excitation with NIR radiation was used as a comparative marker to conclude that specific binding and the ensuing enhanced cellular uptake was clearly more effective when using a targeting ligand on the UCNP surface. Another important finding was regarding the cytotoxicity of the UCNP probes in MTT assays. The works reviewed generally reported that UCNPs did not result in any significant cell death even after 24–48 h incubation times for concentrations ranging from 25 μg/mL to 500 μg/mL (Figure 14) [31,45,46,47,63,65,66,76,115,116,117,118,119,121,122,123]. In contrast, nanoparticles bearing cancer drugs, such as doxorubicin for example, on their surface were found to greatly enhance the death of the cancer cells as would be expected due to the underlying effect of the therapeutic agents (Figure 15).

### 4.1. Animal Imaging

**Figure 16:**
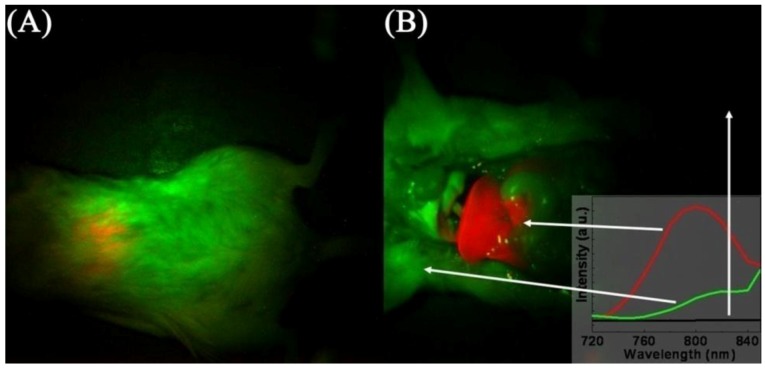
Images of mouse injected *in vivo* with UCNPs; before (**A**) and after dissection (**B**). The red color indicates emission from UCNPs, green and black show background as indicated by the arrows and the inset ([31,125], reproduced by permission of the American Chemical Society).

The majority of papers making use of UCNPs for two-photon excited fluorescence imaging of cancer cells rely on the Er^3+^ emitting ion, which produces upconverted green or red emission upon excitation with 980 nm. However, to improve the contrast and tissue penetration capability of the imaging process, efforts have targeted the use of NIR-to-NIR UCNPs. In fact, Prasad’s group has recently shown that deep tissue imaging can be carried out up to a depth of 3.2 cm [126]. Nyk and co-workers [31] used NaYF_4_:Tm^3+^, Yb^3+^ UCNPs for NIR-to-NIR imaging following a ligand exchange process between the oleate-capping ligand with 3-mercaptopropionic acid. The Tm^3+^ ion has strong emission in the UV/blue and NIR regions. Thus, they exploited the strong NIR emission of Tm^3+^ at approximately 800 nm for NIR-NIR imaging, and so a deep penetration for the excitation light and for observing the emission signal. The authors incubated human pancreatic cancer cells, Panc 1 with UCNPs for 2 h and as a result of complete lack of autofluorescence, high contrast fluorescence images of the cancer cells were obtained. They also noted that the fluorescence imaging showed the feature of inherent three-dimensional localization very similar to images obtained with two-photon excited fluorescence, which is a function of the quasi-quadratic dependence of the emission intensity on the excitation wavelength. Moreover, Nyk and co-workers demonstrated the suitability of this technique for *in vivo* imaging by injecting Balb-c mice and imaging them 2 h post-injection (Figure 16). The authors observed an intense NIR fluorescence centered at approximately 800 nm that was easily detectable through the skin without the removal of the animal’s fur. The animals showed little signs of short-term toxicity up to 48 h post-injection. High contrast images of the liver and spleen were obtained and showed saturated levels of fluorescence, which was indicative of high cellular uptake of the UCNPs. Again, this high contrast was the result of the difference in fluorescence intensity between the background (which was essentially zero) and the fluorescence signal resulting from the NIR-to-NIR upconversion process.

Park *et al*. [51] investigated the bimodal imaging potential of UCNPs by synthesizing 20–41 nm particles. They demonstrated that they can carry out single particle imaging and that these UCNPs can be used for simultaneous optical and magnetic imaging. The work carried out on SK-BR-3 cells showed no autofluorescence and was void of photoblinking (a problem typically associated with some QDs). Imaging was successful even at a concentration of 100 mg/μL Gd^3+^. In addition, at concentrations of ~3.0 mM, both 20 and 41 nm UCNPs showed contrast enhancement in T1-weighted images.

**Figure 17:**
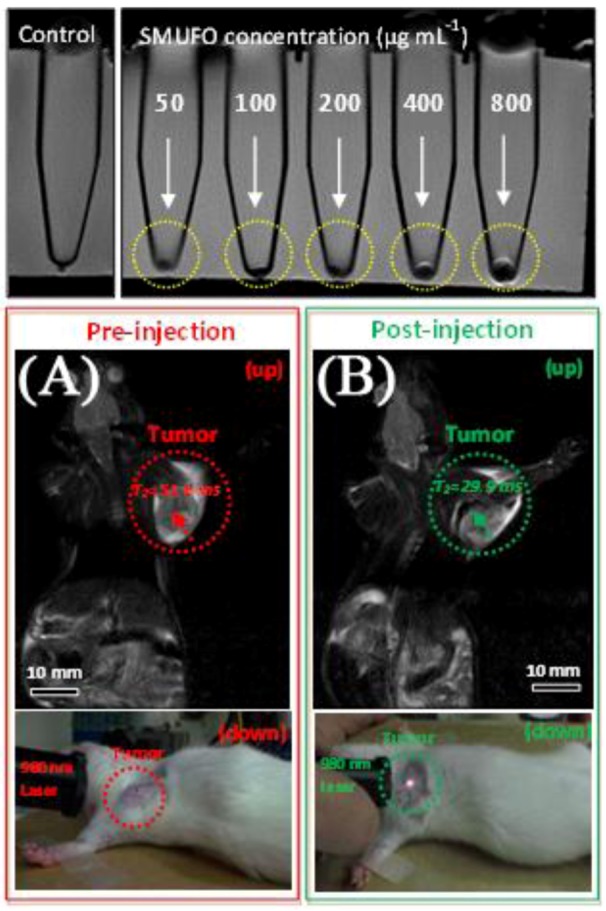
Top: sensitivity of *in vitro* MRI of SMUFO treated MCF-7 cells at different concentrations. Bottom: *in vivo* MR and upconversion fluorescence bimodal imaging of Walker 256 tumor using SMUFO. *In vivo* whole body pre-injection (**A**) and post-injection (**B**) MRI of mouse bearing a Walker 256 tumor. Pre-injection image (A bottom) and post-injection image (B bottom) of the upconversion fluorescence imaging of tumor *in vivo* using NIR laser ([115,125], reproduced by permission of Wiley).

In 2010, Shi and his coworkers [125] prepared bimodal magneto/luminescent probes using the neck formation strategy of silica. A common byproduct of silica coating is the necking behavior of the silica coated particles where two or these particles are conjoined at the edges. The authors prepared superparamagnetic iron oxide nanoparticles (SPIONS) as well as UCNPs co-doped with Er^3+^ and Yb^3+^ and following silica coating of the nanoparticles, they obtained silica-shielded magnetic upconversion fluorescent oligomers (SMUFOs). Enhancement of the contrast was observed in a 3.0T clinical MRI instrument, which prompted *in vivo* studies of the SMUFOs in SD mice bearing a Walker 256 tumor (Figure 17). Following subcutaneous injections, change in the spin-spin relaxation time T_2_ value was observed within 5 min and following NIR excitation of the tumor area, minimal autofluorescence was observed and the tumor was easily observed by the naked eye.

**Figure 18:**
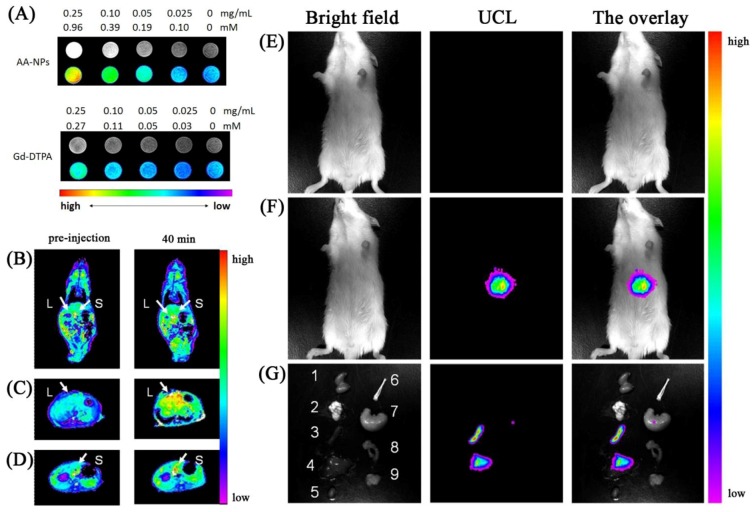
(Left column, **A**): T1-weighted and color-mapped MR images for different mass and molar concentrations of (top) AA-NPs and (bottom) Gd-DTPA. (Left column-bottom): Color-mapped (**B**) coronal images of the whole body and (**C**, **D**) transversal cross-sectional images of the liver (L) and spleen (S) of mice at pre-injection and at 40 min after intravenous injection of AA-NPs. (Right column): *In vivo* imaging of mouse after intravenous injection (**E**) with and (**F**) without AA-NPs. (**G**) images of dissected organs of a mouse sacrificed 40 min after intravenous injection with AA-NPs. 1, kidney; 2, lung; 3, spleen; 4, liver; 5, heart; 6, bone; 7, stomach; 8, intestines; 9, muscle ([117], reproduced by permission of Elsevier).

Li *et al*. prepared tri-doped UCNPs using a hydrothermal method. The particles were rendered hydrophilic *via* oxidation of the capping ligand (oleic acid) [31]. The authors first demonstrated that the particles can be internalized in the cells and do not simply stain the surface. The authors also proceeded to demonstrate that the gadolinium found in the host material may be used as a contrast agent with a similar relaxivity parameter to that reported for Gd-DTPA. Injection in Kunming mice *via* the tail vein resulted in significant contrast enhancement following a comparison of pre and post injection images (Figure 18). *In* and *ex-vivo*, the authors reported that the nanoparticles were highly accumulated in the liver and spleen. Little to no accumulation was observed in the heart, lungs or kidneys. Similar work was reported by Yu *et al*. [45], where they conjugated the nanoparticles with chlorotoxin to target C6 Glioma cells. The authors also carried out *in vivo* studies in Balb-c mice and found no significant change in mice behavior after 7 days. They also found that *ex vivo* analysis of the various organs showed accumulation in the lung, spleen and liver after 7 days. Tumor targeting and imaging in xenograft bearing nude mice was successful and upconversion fluorescence was observed to emanate from the tumor area of the mouse.

**Figure 19:**
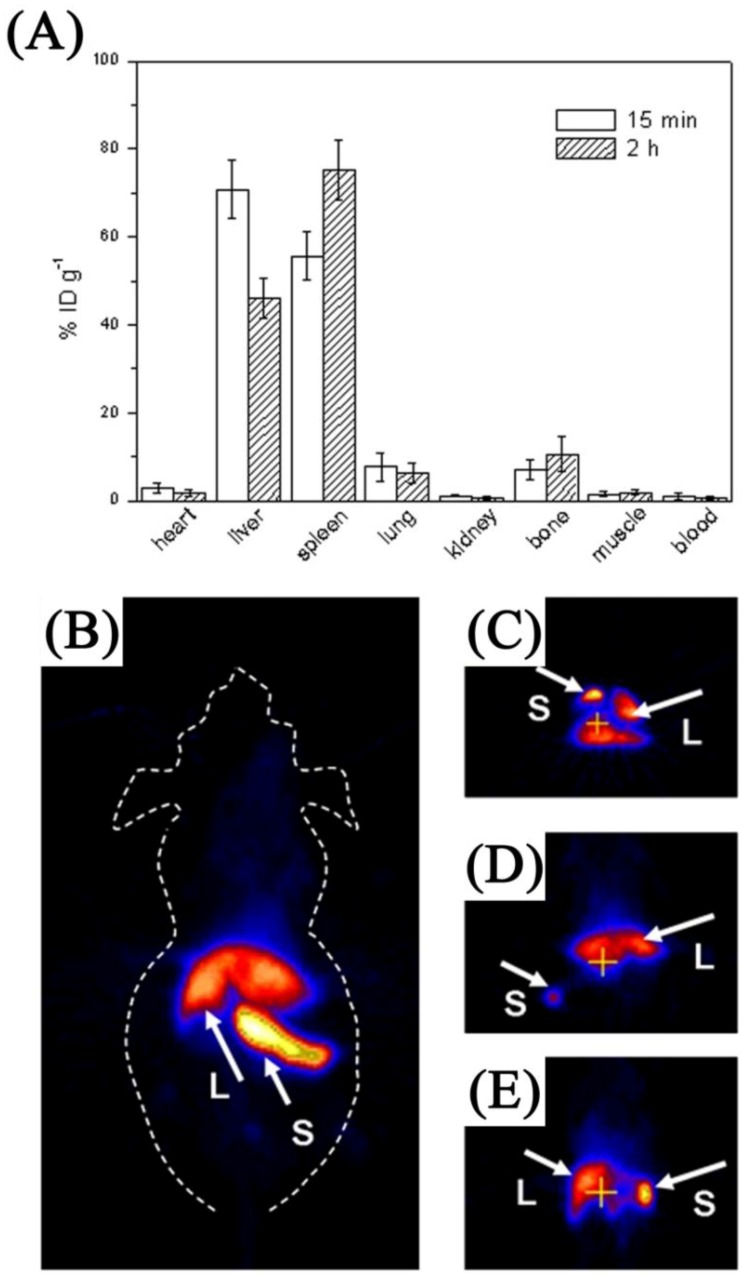
Application of Fluorine labeled UCNPs as multimodal probes (**A**) Biodistribution of at different post-injection times; (**B**) Whole-body two-dimensional projection, and (**C**) transversal, (**D**) coronal, and (**E**) sagittal images of the liver (L) and spleen (S) of an *in vivo* injected mouse ([125], reproduced by permission of Elsevier).

One instance of multimodal imaging using MRI and PET was reported by Zhou *et al*. [117]. The authors prepared Na(Gd)YF_4_ nanoparticles co-doped with Er^3+^ and Yb^3+^. Incubation with KB cells for even 1 h showed that the UCNPs penetrated the cells and bright upconversion luminescence was observed following 980 nm excitation. Strong contrast brightening was observed as the concentration of the Gd^3+^ ion increased in T_1_ weighted MR imaging. Pre and post *in vivo* MRI showed contrast enhancement in the spleen and liver after 15 min injection of the UCNP system. Preparation of 18F labeled UCNPs allowed for simultaneous MRI and PET imaging. Fifteen minutes post injection, intense radioactive signal were observed exclusively in the spleen and liver (similar to MRI contrast enhancement following the same time scale). The authors reported that low uptake of UCNPs was observed in the heart, lung and kidneys, which was similar to previous reports discussed above (Figure 19).

Imaging of the organs was reported by Cheng *et al*. [110] where 3 UCNP systems were prepared each one doped with a different Ln^3+^ ion with a specific upconversion emission signature (λ_exc_ = 980 nm). The authors were interested in multiplexed *in vivo* imaging of the lymphatic network in the neck and upper trunk of nude mice. Imaging of the mice pre and post-mortem showed that two lymph nodes could be identified using different upconversion emission wavelengths suggesting the high potential for using such a system for lymph node imaging. In addition the authors carried out *in vivo* cell tracking studies using KB cells. The authors incubated the cells with the UCNPs and following subcutaneous injection in mice and imaging, they were able to track the incubated cells. This was the first instance where color multiplex UCNP imaging was reported. The authors also demonstrated the imaging sensitivity of the UCNP system relative to QDs and showed that the detection power of UCNPs was one order of magnitude better than that of the QD systems due to the absence of autofluoresence.

### 4.2. Imaging and Photodynamic Therapy

UCNPs have been shown to be more than just bioprobes for upconversion fluorescence imaging of cancer. In fact, the notion of using upconverting sensitizers in PDT was discussed in the early 1990’s [127,128,129,130]. Chatterjee *et al*. [118] demonstrated that folic acid (FA) conjugated NaYF_4_:Er^3+^, Yb^3+^ UCNPs could be used in photodynamic therapy (PDT) applications when in the vicinity of the photosensitive dye zinc phthalocyanine (ZnPC). In this paper, the authors physically adsorbed the ZnPC dye to the nanoparticle surface and tested its singlet oxygen production when in the presence of a molecular probe (ADPA, anthracene-9, 10-dipropionic acid), which is destroyed in the presence of the singlet oxygen species. This was accomplished by monitoring the absorption of ADPA at 400 nm following excitation with 980 nm NIR light and the results showed excellent effectiveness in generating singlet oxygen. Subsequently, the authors tested the FA-UCNP/ZnPC nanostructure in the presence of human colonic adenocarcinoma cells (HT29 cell line), which are known to overexpress folate receptors to determine their efficacy in photodynamic therapy. The authors showed that the cell viability decreased drastically when excited for 30 min at 980 nm. This was the first demonstration of the NIR excited UCNP being used in photodynamic therapy applications.

**Figure 20:**
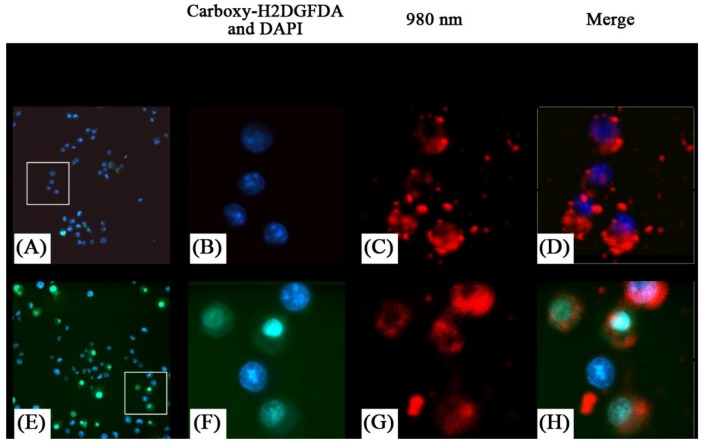
Detection of oxidative stress in live cells using MB49-PSA cells treated with NaYF_4_ nanoparticles (**A–D**) or ZnPc-loaded NaYF_4_ nanoparticles (**E–H**) followed by laser activation to induce oxidative stress. Insets in (**A**) and (**E**) show the region that has been enlarged in (**B–D**) and (**F–H**) respectively. Presence of reduced oxygen species are shown in green, (green) nuclei in blue, and the nanoparticles are shown by their red fluorescence color ([76], reproduced by permission of Elsevier).

Similarly Guo *et al*. [76] investigated the use of UCNPs with ZnPc for use in PDT therapy. The PDT drug was loaded in the pores of a mesoporous silica coating the UCNPs. The authors reported that significant singlet oxygen generation was obtained following irradiation with 980 nm exciting the UCNPs and energy transferring to the ZnPc (Figure 20). This was observed in MB49 PSA cells as well as in MTT assays where cell viability decreased significantly in ZnPc loaded UCNP systems relative to ZnPc alone. The secretion of PSA from MB49PSA allows the use of the former as a tumor marker. A 30% decrease in PSA levels was measured in ZnPC-loaded UCNPs incubated cells due to the efficient cell death brought about by the UCNP system.

## 5. Luminescent UCNPs as Nanothermometers

Up to this point, it was discussed that Ln^3+^-doped UCNPs are extremely useful for high-resolution fluorescence imaging of cancer. It has also been recently shown that fluorescent nanoprobes (nanocrystals, semiconductor QDs, nanogels, *etc*.) can be used for thermal sensing, *i.e*., for determining temperature gradients and temperature changes with a potential spatial resolution at the nanoscale and so at the cellular level [52,131,132,133,134,135,136]. These temperature sensitive fluorescent nanoprobes are now commonly referred to as “fluorescent nanothermometers”. The discovery of these nanothermometers could be of particular relevance for cancer therapy in order to control and understand the hyperthermia processes applied for the selective treatment of malignant cells [137,138,139]. Moreover, intracellular thermal measurements could be used as a non-invasive diagnostic tool by taking advantage of the temperature differences that seem to occur between healthy and pathological cells [140,141]. In this section we describe how UCNPs can be used for nanothermometry by invoking the particular case of the recently reported NaYF_4_:Yb^3+^,Er^3+^ upconverting nanothermometers [52].

The principle behind temperature measurement using a fluorophore (*i.e*., a fluorescent material) is rather simple. It is essentially based on the temperature sensitivity of one or more of the fluorophore emission features; such as emission intensity, shape, peak position or lifetime. Thus, knowing the temperature dependence of the spectral and/or temporal emission implies that the temperature of the investigated system can be determined. One of the main advantages of this method is its non-contact nature, which provides minimal perturbation of the system under investigation. Indeed this advantage is essential for temperature measurements in fluids and particularly in biological systems. For these systems, multi-photon NIR-excited upconverting fluorophores are strongly preferred, so that autofluorescence (*i.e*., endogenous fluorescence of the system) and photo-bleaching (*i.e*., damage by ultraviolet-visible excitation light) are strongly reduced. In addition, the multi-photon excitation nature leads to an increased spatial resolution [23]. At present two types of nanoparticles have been reported to operate as two-photon excited fluorescent nanothermometers; CdSe and CdTe quantum dots [131], as well as NaYF_4_:Yb^3+^, Er^3+^ nanoparticles [52]. These nanoparticles produce visible luminescence under two-photon excitation and can be easily internalized by the cells [64] allowing for intracellular temperature measurements. Moreover, this approach allows also for cell imaging by means of two-photon excited fluorescence microscopy. Indeed these fluorescent nanoparticles or “nanothermometers” provide the dual functionality of imaging and temperature sensing allowing for high spatial resolution intracellular thermal imaging. In fact, the actual resolution is limited by the Rayleigh limit in far-field fluorescence microscopes (~200 nm) and could be even reduced in near field fluorescence microscopes (~50 nm). These resolutions are considered high compared to the typical cell sizes of approximately tens of microns.

**Figure 21:**
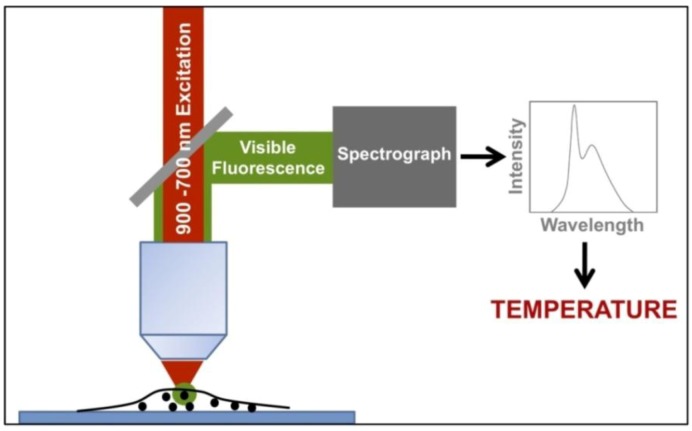
Typical experimental arrangement for measuring intracellular temperatures by means of two-photon excited fluorescent nanoparticles.

Figure 21 shows the typical experimental arrangement for measuring intracellular temperatures by means of two-photon excited fluorescent nanoparticles. A NIR excitation tunable laser beam, tuned at the appropriate excitation wavelength, is focused onto the cell. The visible light emitted from the nanoparticles is directed to a spectrometer by means of a suitable dichroic mirror, which reflects only the visible fluorescence but not the NIR excitation light scattered by the cell. Thus, the temperature is determined in different points inside the cell by scanning the excitation beam across the cell while registering the emission spectrum given by the nanoparticles at each one of the selected points. It is important to mention that the NIR excitation laser wavelength must be selected to excite only the fluorescent nanoparticle while not interacting with the cell fluids. Otherwise, the cell can be heated by the excitation light, for instance by water absorption. Moreover, the input intensity must be low enough in order to minimize any light induced heating due to non-radiative de-excitations of the excited fluorescent nanoparticle, which could lead to undesired temperature alterations [142].

**Figure 22:**
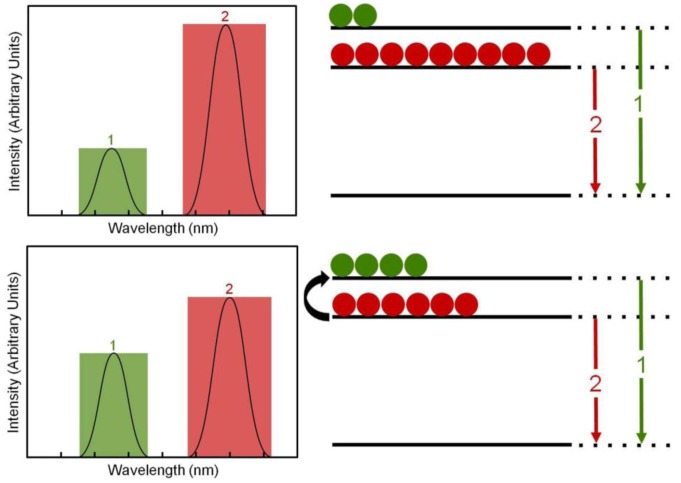
Temperature estimation mechanism based on Ln^3+^ doped nanoparticles.

The “nanothermometers” based on Ln^3+^-doped nanoparticles use two excited electronic energy states that, when de-exciting, produce two close emission bands, denoted by 1 and 2 in Figure 22. These exited states are so closely spaced that their populations, *N*_1_ and *N*_2_, are very sensitive to the environmental temperature by a well-known thermal equilibrium equation: 


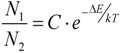
(1)

where C is a constant for each particular ion-host system, Δ*E* is the energy gap between the two excited states, *k* = 8.62 × 10^-5^*eVK*^-1^ is the Boltzmann constant and *T* the absolute temperature. Thus, an increase in temperature produces a corresponding increase in the relative population *N*_1_/*N*_2_ and so in the relative emission intensity *I*_1_/*I*_2_ ≈ *N*_1_/*N*_2_ (where *I*_1_ and *I*_2_ are the intensities of the emissions labeled as 1 and 2 in Figure 22. As a result the temperature can be solved from equation (1) and so estimated if the emission intensity ratio *I*_1_/*I*_2_ is measured and Δ*E* and *C* are known. Usually the intensity *I*_1_/*I*_2_ ratio is previously calibrated as a function of temperature and a thermometric scale is obtained, so that a direct measure of the cell temperature is measured from the Ln^3+^-doped nanoparticle emission spectrum.

At the present moment, nanothermometers based on NaYF_4_:Yb^3+^,Er^3+^ nanoparticles have been already reported [52]. In these nanothermometers, suitable excited states (labeled as ^2^H_11/2_ and ^2^S_3/2_) of Er^3+^ ions are used to produce two green emission bands centered at around 525 nm and 545 nm. The relative intensity of these emission bands *I*_1_/*I*_2_ = *I*_525_/*I*_545_ has been reported to be quite sensitive to temperature and so it has been used to measure absolute temperatures by means of a previously established calibration expression:


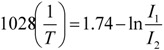
(2)

where T is given in K. In these nanothermometers Er^3+^ ion act as a temperature sensitive emitting ion while Yb^3+^ is used as an efficient NIR absorbing ion that populates the exited states of the emitting Er^3+^ ion *via* upconversion (Yb^3+^ → Er^3+^ energy transfer). Thus a two-photon NIR excited green emitting NaYF_4_:Yb^3+^, Er^3+^ nanothermometer was developed, which was successfully used to image temperature gradients in liquids. More interestingly from the perspective of this work is that these nanoparticles were used for detecting intracellular temperature changes externally induced within HeLa cancer cells. In this case the cancer cells were heated by means of a metallic platform connected to a resistor in contact with the cell bath. By changing the applied voltage, the temperature was increased from about 25 °C to about 45 °C, where a small cell fragmentation started to be observed.

**Figure 23:**
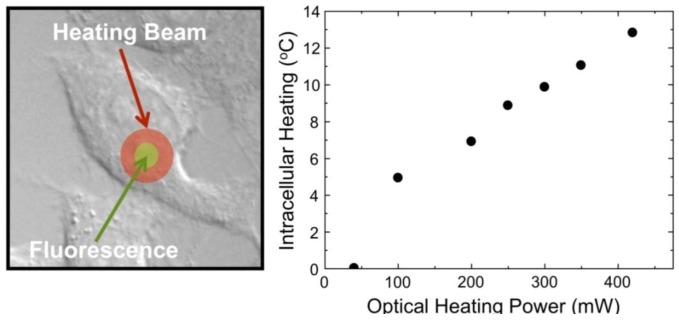
Intracellular temperature increase as a function of the heating beam power.

Figure 23 demonstrates the ability of the NaYF_4_:Yb^3+^, Er^3+^ nanothermometers for measuring intracellular temperature changes within an individual HeLa cervical cancer cell. In this experiment, the cell was warmed by a laser beam at 980 nm (the heating beam) that illuminates the broad area marked in red. This beam is absorbed by the cellular fluid and produces a light induced heating. Then the temperature is measured within the heated area by exciting the Yb^3+^ ion (at the non-heating 920 nm wavelength) and recording the Er^3+^ green emission, and so the *I*_1_/*I*_2_ ratio, from which the temperature is determined. The right side in Figure 23 shows the intracellular temperature increase as a function of the heating beam power. By increasing the power of this beam up to 400 mW a temperature increase of about 14 °C is reached. This “non-contact” heating is not relevant enough to produce morphological changes in the HeLa cell but manifests the ability of the NaYF_4_:Yb^3+^, Er^3+^ nanoparticles as intracellular nanothermometers within an accuracy of about 1 °C. Additional experiments are currently underway to improve the above-mentioned sensitivity by using other Ln^3+^ emitting ions.

## 6. Conclusions and Future Outlook

Nanosystems have several important properties that distinguish them from other cancer therapeutic approaches since they can be designed and tailored for predetermined functionalities including: detection (through imaging), targeting (functionalization of one or more ligands), and therapeutics (nanoparticles or nanoparticle-drug conjugates). New synthetic methods have been developed to precisely control the size, shape and composition of the nanoparticles in order to tune absorption and emission properties for high contrast imaging agents [142]. In this respect, the novel upconverting nanoparticles discussed herein present promising perspectives. As their absorption/emission properties are unaffected by size and shape and so by the inevitable size and shape dispersion, a typical problem associated to quantum and metallic nanoparticles. Moreover it does point to the dominant efficiency of the lanthanide doped nanoparticles with respect to these commercial nanoparticles, which opens new avenues for cheaper imaging technologies. The multimodal possibilities of the upconverting nanoparticles have been also here manifested and make them even more attractive not only as passive nanofluorophores but also as active nanofluorophores agents.

In this respect, nowadays nanocarriers are being developed for drug delivery purposes and for increasing intracellular concentration of drugs in cancer cells, while minimizing toxicity in normal cells; simultaneously enhancing anti-cancer effects and reducing systemic toxicity [3,143]. Currently, two therapeutic nanocarrier-liposomes and albumin nanoparticle systems have already been approved by the USFDA for clinical practices [144]. In addition, nanoparticles can be used to target specific sites *via* surface modifications for providing specific biochemical interactions with the receptors of target cells as well as to deliver drugs to the target site, crossing several biological barriers. Some examples of these strategies have already been published by numerous research groups and has evaluated coating nanoparticles with polysorbates where the drug-loaded nanoparticles can be transported across the blood–brain barrier [145,146,147]; *via* coating with epithelial growth factor antibody-conjugated rapamycin-loaded nanoparticles showing an enhancing efficacy in MCF7 breast cancer-cell line [143], or finally by conjugating nanoparticles to a nuclear localization sequence for the direct targeting of doxorubicin to the nucleus of breast cancer cells [148]. Thus, the number of possibilities for the novel upconverting lanthanide nanoparticles, which we have shown to present an easy management of functionalization, is considerably increased.

Apart from these possibilities, nanotechnology is also now being applied towards the development of relatively novel cancer therapies, including, gene therapy, photodynamic therapy (based in the combination of a photosensitizer, light and oxygen), radiotherapy and radiofrequency, as well as cancer theragnosis (combination of diagnosis and therapy) [149,150,151,152]. Thus, the development of upconverting nanoparticles as imaging contrast agents with a capacity for targeting tumours and also delivery of therapeutic agents together with the possibility of intracellular temperature determination provides a promising scenario for these multifunctional nanoparticles. In fact it does open the possibility of development of multifunctional nanoparticles to detect and kill cancer cells simultaneously and therefore, providing opportunities for designing properties that are not possible with other conventional types of therapeutic drugs. In essence we envisage bright future for these novel nanoparticles to support a new generation of cancer therapeutics. Nevertheless certain critical questions remain and must be addressed prior to the clinical development of any kind of nanoparticle for use in diagnostics and therapeutics in cancer (and other diseases). The current knowledge regarding the safety of nanoparticles is insufficient and speaks to the necessity for analysis of the pharmacokinetic behavior of different types of nanoparticles and the distribution and accumulation in tissue systems. This in itself, is not a trivial matter as many parameters will affect the toxicity and tissue localization of the nanoparticles including size, morphology, capping ligand(s), phase, crystallinity, and surface charge to name a few.

In summary, the application of nanotechnology in cancer has experienced an exponential growth in the past few years. Nanoparticles provide opportunities for specific design and present fascinating properties that are not possible with other drugs typically used in cancer therapies. Future studies will likely focus on active targeting with molecules for both specific recognition and cell internalization. Ultimately, this will lead to the development of a vehicle, which will be capable of targeting, imaging, diagnostics and therapeutics. Thus, there is no doubt that the development of these novel lanthanide doped nanoparticles will play a significant role in the near future.
